# Neighborhood Physical Disadvantage, Education, and Cognitive Decline Trajectories in U.S. Older Adults

**DOI:** 10.1093/geroni/igaf122.1364

**Published:** 2025-12-31

**Authors:** Kedong Ding

**Affiliations:** Case Western Reserve University, Cleveland, Ohio, United States

## Abstract

One in four U.S. older adults experiences cognitive decline before death, which can significantly affect their quality of life and independence. Given the important role of neighborhood environments in cognitive health, it remains unclear whether living in physically disadvantaged neighborhoods has a long-term impact on cognitive trajectories. This study aims to identify the trajectories of cognitive decline and examine the associations between the trajectories and neighborhood physical disorder, and whether these associations differ by educational background. We used data from 7,460 participants aged 50+ in the Health and Retirement Study (HRS) from 2006 to 2020. We used group-based trajectory models to identify latent groups for trajectories of cognitive decline over time. Multinomial logistic analyses were adjusted for sociodemographic and health covariates. We identified five trajectory groups: Group One (“high start, stable”), Group Two (“medium-high start, stable”), Group Three (“medium-high start, slight decrease”), Group Four (“medium start, sharp decrease”), and Group Five (“low start, slight decrease”). We found that neighborhood physical disadvantage was associated with higher odds of belonging to Group One (RRR=1.17, P < 0.01), Two (RRR=1.28, p < 0.001), Three (RRR=1.33, p<.001) and Four (RRR=1.23, p < 0.01), all with lower baseline cognition and faster decline than Group One, which had the best cognitive trajectory. Additionally, participants with more than a high school education who lived in disadvantaged neighborhoods were more likely to be in groups with lower baseline cognition and faster decline than Group One. Our findings highlighted the important role of neighborhood physical disadvantage and education in older adults’ cognitive function trajectories.

